# Reduced Theta-Band Power and Phase Synchrony during Explicit Verbal Memory Tasks in Female, Non-Clinical Individuals with Schizotypal Traits

**DOI:** 10.1371/journal.pone.0148272

**Published:** 2016-02-03

**Authors:** Jeong Woo Choi, Kyoung-Mi Jang, Ki-Young Jung, Myung-Sun Kim, Kyung Hwan Kim

**Affiliations:** 1 Department of Biomedical Engineering, College of Health Science, Yonsei University, Wonju, Gangwon-do, Republic of Korea; 2 Department of Psychology, Sungshin Woman’s University, Seoul, Republic of Korea; 3 Department of Neurology, Seoul National University College of Medicine, Seoul, Republic of Korea; Center for BrainHealth, University of Texas at Dallas, UNITED STATES

## Abstract

The study of non-clinical individuals with schizotypal traits has been considered to provide a promising endophenotypic approach to understanding schizophrenia, because schizophrenia is highly heterogeneous, and a number of confounding factors may affect neuropsychological performance. Here, we investigated whether deficits in explicit verbal memory in individuals with schizotypal traits are associated with abnormalities in the local and inter-regional synchrony of brain activity. Memory deficits have been recognized as a core problem in schizophrenia, and previous studies have consistently shown explicit verbal memory impairment in schizophrenic patients. However, the mechanism of this impairment has not been fully revealed. Seventeen individuals with schizotypal traits and 17 age-matched, normal controls participated. Multichannel event-related electroencephalograms (EEGs) were recorded while the subjects performed a continuous recognition task. Event-related spectral perturbations (ERSPs) and inter-regional theta-band phase locking values (TPLVs) were investigated to determine the differences in local and global neural synchrony between the two subject groups. Additionally, the connection patterns of the TPLVs were quantitatively analyzed using graph theory measures. An old/new effect was found in the induced theta-band ERSP in both groups. However, the difference between the old and new was larger in normal controls than in schizotypal trait group. The tendency of elevated old/new effect in normal controls was observed in anterior-posterior theta-band phase synchrony as well. Our results suggest that explicit memory deficits observed in schizophrenia patients can also be found in non-clinical individuals with psychometrically defined schizotypal traits.

## Introduction

Memory deficits have been recognized as a core problem in schizophrenia [1, 2]. In particular, explicit verbal memory dysfunction in schizophrenic patients has attracted considerable interest and has provided evidence for the role of the medial temporal lobe (MTL) and prefrontal cortex (PFC) in explicit verbal memory [3, 4]. Evidence of abnormalities in these structures in schizophrenic patients has been accumulating [5–8]. Furthermore, recent neuroimaging studies have reported an association between explicit verbal memory impairments and structural/functional abnormalities in left medial temporal areas in schizophrenic patients [9–11]. For example, van Erp et al. (2008) observed significant correlations between left hippocampal volume and performance on the California Verbal Learning Test in schizophrenic patients.

Schizophrenia is highly heterogeneous, and a number of confounding factors, such as antipsychotic drugs and the length of illness and hospitalization, may affect neuropsychological performance. Thus, the study of patients with schizotypal personality disorder and non-clinical individuals with schizotypal traits has been considered to provide a promising endophenotypic approach to understanding schizophrenia [12]. In particular, studies of non-clinical individuals with psychometrically defined schizotypal traits have drawn attention because both neuroimaging and neuropsychological evidence support the idea that schizotypy in healthy populations and schizophrenia are fundamentally related [13]. For example, non-clinical individuals who obtained high scores on the Schizotypal Personality Questionnaire (SPQ) [14] exhibited reduced gray matter density in the insula and dorsolateral prefrontal gyrus and weaker functional connectivity between regions within the medial frontal gyrus than those who obtained low scores on the SPQ [15]. In addition, a significant negative association between activation in the left/right middle temporal gyrus and the SPQ score and a positive correlation between activation in the left inferior frontal gyrus and the SPQ score were observed during a reading task. Additionally, compared with those who obtained low scores, individuals who obtained high SPQ scores showed cognitive deficits, including executive function [16, 17] and conscious memory [18]. Based on previous studies suggesting that nonclinical schizotypy is not affected by the confounding factors, and subtle cognitive abnormalities precede the first psychotic episode [19], we anticipated that an investigation on the memory function of nonclinical individuals with schizotypal traits would provide valuable insight into the neuropathology underlying schizophrenia, and we have been trying to elucidate its neural basis [20]. Here, we tried to investigate whether the deficits in explicit verbal memory function associated with schizotypal traits are due to abnormal cortical rhythm.

Although previous studies have consistently shown explicit verbal memory impairment in schizophrenic patients, the mechanism of this impairment has not been fully revealed due to the complicated processing of memory, which consists of multiple components, such as encoding, storage, and retrieval. In fact, some studies have reported that memory impairment in schizophrenic patients is due to an encoding deficit [21], a retrieval problem [22], or both [23]. Others have insisted that the memory impairment of these patients is related to rapid forgetting [24].

Event-related potentials (ERPs), the electrical recordings of brain activity that are time-locked to external events, have been widely used to investigate memory function due to the high temporal resolution of this technique. Numerous studies have shown that ERPs elicited by repeated items (old) are generally more positive-going than ERPs elicited by new items (new); this phenomenon has been referred to as the old/new or repetition effect. The old/new effect is known to begin at approximately 250–300 ms and to last until 700–800 ms after stimulus onset [25]. A number of ERP studies of explicit verbal memory in schizophrenic patients have reported a reduction in the old/new effect, i.e., reduced ERP amplitude differences between new and old stimuli [26–28]. For example, Baving et al. (2000) observed a smaller old/new effect in the parietal region in schizophrenic patients than in normal controls 250–400 ms after stimulus onset. Kayser et al. (2009) found a significantly lower old/new effect in lateral temporoparietal sites in schizophrenic patients. Similarly, in a recent ERP study, we observed explicit verbal memory deficits in college students with schizotypal traits [20]. We investigated explicit verbal memory using a continuous recognition task, and compared with normal controls, individuals with schizotypal traits exhibited a significantly lower old/new effect 550–650 ms after stimulus onset. During this period, significantly stronger ERP amplitudes for old words than for new words were observed only in the normal controls. The dual-process model of recognition memory suggests that recognition memory consists of the earlier assessment of stimulus familiarity and the later recollection of the details associated with a previous episode [29, 30]. Based on this model, we concluded that recollection capabilities during the later period (550–650 ms here) were lower in individuals with schizotypal traits [20].

Beyond conventional averaged ERPs, the analysis of neural oscillations reflecting the synchrony of local neuronal activity has drawn significant attention in neurocognitive studies using electroencephalogram (EEG) and magnetoencephalogram (MEG) recordings. Enhancement of the oscillatory rhythm in the theta band (4–8 Hz) has been observed during memory tasks in a number of EEG [31] and MEG studies [32]. For instance, Gevins et al. (1997) reported a significant correlation between theta-band power and working memory (WM) load; i.e., theta-band rhythm increased in proportion to increased WM load. Prominent theta rhythms (recorded by MEG) were found in the MTL and PFC [32] during the encoding and retrieval of explicit memory. The enhancement of theta-band power in local field potentials of the rodent hippocampus was observed during the maintenance condition of a WM task [33]. In addition, inter-regional synchrony of neural oscillations, which signifies the functional integration of multiple neuronal assemblies [34, 35], has been investigated in various cognitive functions, including memory. Coherence between the theta-band rhythms of the frontal and posterior regions was observed during WM tasks [36]. Phase synchrony between frontal and posterior theta rhythms was observed during successful information encoding [37, 38]. In line with these results from normal subjects and with the putative role of theta rhythm in memory and cognitive control, a reduction in theta power during memory functions was observed in patients with schizophrenia [39]. Notably, neither an increase in theta power during WM tasks nor a theta power-WM load correlation was observed in schizophrenic patients, contrary to those observed in normal controls. Additionally, frontal-temporal coherence in the theta band during talking was significantly diminished in schizophrenic patients [40].

The purpose of the current study was to investigate whether the deficits in explicit verbal memory observed in individuals with schizotypal traits in our previous study are associated with oscillation abnormalities and the inter-regional synchrony of brain activity. Specifically, we expected that the observation of a reduced old/new effect in individuals with schizotypal traits would be accompanied by a reduced old/new effect in theta-band oscillations and inter-regional synchrony.

## Materials and Methods

### Participants

The characteristics of the participants employed in this study were presented in our previous study [20]. A total of 34 female college students without any history of psychiatric or neurological disorders were recruited from a pool of 610 students based on their scores on the Korean version of the SPQ [41]. The schizotypal trait group (n = 17) was composed of the individuals who obtained the highest 3% of the distribution of scores (score range: 36–53), and the control group (n = 17) consisted of those who obtained average scores (score range: 14–23) on the SPQ. The two groups did not differ in age (20.7±1.53 years for the schizotypal trait group, 20.07±2.02 years for the control group, *F*(1,32) = 1.20, *p* = 0.317) or duration of education (14.20±1.86 years for the schizotypal trait group, 13.07±1.98 years for the control group, *F*(1,32) = 1.61, *p* = 0.117). The SPQ scores of the schizotypal trait group were significantly higher than those of the control group (42.35±6.47 for the schizotypal trait group, 17.29±3.72 for the control group, *F*(1,32) = 191.61, *p*<0.001).

All participants were right-handed, and none of the participants were taking medication at the time of testing. Written informed consent was obtained from all participants after they were provided with a complete description of the study. The study was approved by the Sungshin Women’s University Institutional Bioethics Review Board.

### The continuous recognition task

The continuous recognition task used to measure explicit verbal memory function was described in our previous study [20]. A total of 380 Korean words (nouns for animals and plants) were used as stimuli. These stimuli were arranged into two blocks of trials. In each block, 50 words were presented only once (new), and 140 words were repeated following one to five intervening words (old). The participants continuously viewed a series of words and were required to press one of the two response buttons according to the type of word presented (old or new).

The stimuli were presented on a computer monitor within foveal vision for 200 ms and were subtended at a vertical visual angle of 2.29° and a horizontal visual angle of 3.43°. A crosshair (+) was displayed on the screen for 500 ms as a fixation point prior to the presentation of each stimulus. The inter-stimulus interval was 2000 ms. A block of 10 practice trials was administered prior to the experimental session.

### Electrophysiological recording procedures

EEGs were recorded using a 64-channel Geodesic Sensor Net connected to a 64-channel amplifier (Net Amp 300: Electrical Geodesics, Eugene, OR, USA). Each electrode was referenced to Cz and adjusted until the impedances were less than 50 kΩ [42]. Eye movements and blinks were monitored with electrodes placed near the outer cantus and beneath the left eye.

The EEGs were recorded using a 0.1–100 Hz analog bandpass filter and a sampling rate of 250 Hz. After the completion of data collection, the EEGs were segmented into 1500 ms epochs, including a 500 ms prestimulus baseline with respect to stimulus onset. The epochs contaminated by artifacts, such as eye blinks and eye movements, were excluded from further analysis. (The threshold for artifact rejection was ± a peak-to-peak amplitude of 70 μV.) An average-reference transformation was used to minimize the effects of reference site activity [43].

### Event-related spectral perturbation (ERSP)

To examine the temporal evolution of spectral characteristics by ERSP, a continuous wavelet transform (CWT) using the Morlet wavelet was applied to the data of each single trial as described in [44] (frequency range from 1 to 100 Hz, in 1 Hz steps). The number of cycles for the CWT linearly increased according to frequency: from 4 at the lowest frequency (1 Hz) to 8.75 at the highest frequency (100 Hz; see [45]). This method provides better frequency resolution at high frequency, and it is better matched to the linear scale adopted for the visualization of time-frequency maps [45]. Induced (i.e., non-phase locked) theta-band activity (TBA) was obtained by averaging the ERSP patterns from each single trial [46].

The ERSP map of each single trial was normalized using surrogate ERSP maps obtained from 500 surrogate data that were obtained by adding random phases to each original single trial in the frequency domain so that the phase was randomized but the amplitude was preserved, as illustrated in [47]. The mean and standard deviation (SD) within each specific time-frequency window were calculated from the ERSP maps of the 500 surrogate data points. The normalized ERSP map was obtained by subtracting the mean ERSP map from the original ERSP map and then dividing it by the SD.

### Theta-band phase locking value (TPLV)

Inter-regional phase synchrony in the theta band was quantified by calculating the theta band phase locking value (TPLV) [34]. Single-trial ERP signals were first transformed into narrowband signals in the theta band through bandpass filtering (4–8 Hz). The instantaneous phase *ϕ*(*t*) for each time point *t* was calculated from the narrowband signal *x*(*t*) and its Hilbert transform as follows [34, 46]:

$$\phi (t)=\text{arctan}\frac{\tilde{x}(t)}{x(t)}$$ (1)

The PLV between two electrodes, *j* and *k*, was calculated for each time point, *t*, by averaging the phase difference over *N* trials as follows [34]:

$$PLVj,k,t=\frac{1}{N}\left|\sum_{n=1}^{N}\text{exp}[i\{\phi_{j}(t,n)-\phi_{k}(t,n)\}]\right|$$ (2)

Here, *N* represents the total number of trials, and *n* denotes a specific trial. *ϕ*_*j*_(*t*, *n*) designates the phase of the signals from electrode *j* at time *t* of the *n*^*th*^ trial.

We selected only 20 non-adjacent electrodes out of 64 (Fp1, Fp2, F7, F3, Fz, F4, F8, C3, Cz, C4, T3, T4, T5, P3, Pz, P4, T6, O1, Oz, and O2). The selected electrodes were distributed sparsely in space to alleviate volume conduction problems among neighboring electrodes [34]. We also verified that the electrode pairs located close together showed the tendency to yield a less significant connection. To identify electrode pairs with significant phase synchrony, we used a double-threshold strategy based on two criteria [48]. The first step involved checking whether the PLV change under investigation was meaningful with respect to the PLVs of the surrogate data, which were obtained from random trial shuffling [34]. The surrogate data were obtained from 200 random trial shuffles for each electrode pair. Using the distribution of the PLVs calculated from the surrogate data, the significance level could be obtained as explained by [34, 49]. The significance level was set to 5%; i.e., the phase synchrony was determined to be significantly increased if it was higher than the fifth percentile of the PLVs of the surrogate data. The second criterion was to determine whether the PLV increased significantly during task execution compared to the prestimulus baseline PLV. After obtaining the baseline distribution of PLVs during the 300 ms period (75 samples) before stimulus onset, the level of significance was set to 5%, the same as that of the first step. In other words, we determined whether phase synchrony grew significantly higher than the fifth percentile of the PLVs obtained from the EEG during the prestimulus period.

### Graph theory analysis of the phase synchrony maps

The spatial pattern of inter-regional phase synchrony can be visualized as a graph consisting of nodes and edges. Thus, the spatial pattern can be quantitatively analyzed using graph theory measures [50]. The nodes of the graph are determined by the electrodes included in the phase synchrony analysis, and the edges are determined by the electrode pairs showing significant phase synchrony. The graph can be quantitatively characterized by a symmetric adjacency matrix. The undirected binary element a_*ij*_ of the adjacency matrix represents a connection between nodes *i* and *j*, and it is designated as 1 or 0 depending on the presence or absence of a significant connection between the two nodes.

A set of numerical measures describing the properties of the graph can be derived from the adjacency matrix [50]. The clustering coefficient, *C*, is used as an index of local connectivity and is defined by the following equation:

$$C=\langle c\rangle =\frac{1}{N}\sum_{i=1}^{N}c_{i}$$ (3)

where *c*_*i*_ is the ratio of the number of existing edges between neighbors, which are connected to electrode *i* by an edge. The maximum possible number of edges between neighbors is represented by *i*. *C* can be interpreted as a measure of the network’s resilience to error because in the case of high clustering, even if a node is lost, its neighbors remain connected.

In addition to the measure of local connectedness, the characteristic path length, *L*, can provide an important measure to characterize global connectedness and is defined as follows:

$$L=\frac{1}{N(N-1)}\sum_{i,j\in N,i\neq j}d_{i,j}$$ (4)

where the path length, and *d*_*i*, *j*_ is the minimum number of edges that must be traversed to go from node *i* to node *j*. Because *L* is the average path length between all possible pairs of *N* nodes, it represents global connectivity, which describes how well integrated a graph is and how easy and fast it is to transport information in the network [51]. The Brain Connectivity Toolbox (http://www.brain-connectivity-toolbox.net), an open-access MATLAB network analysis toolbox, was used to calculate these graph theory measures.

To determine whether the TPLV pattern corresponded to a ‘small-world network’ [52], which is known to be the optimal structure for inter-regional communication [53, 54], we applied the method of [55]. The network small-worldness, *S*, is computed by the ratio of the normalized clustering coefficient (*C*_*norm*_) and the characteristic path length (*L*_*norm*_) as follows:

$$S=\frac{C_{norm}}{L_{norm}}=\frac{C/C_{rand}}{L/L_{rand}}$$ (5)

where *C*_*rand*_ and *L*_*rand*_ are the *C* and *L* derived from random networks, respectively. One hundred random networks with identical degree distributions were generated as the experimental TPLV network for each subject to calculate *C*_*rand*_ and *L*_*rand*_.

Graph theory measures are dependent on the number of edges in each graph; thus, they may be significantly affected by the number of connections rather than by the spatial pattern of the graph itself. This problem can be alleviated by adjusting the number of significant connections so that the number is identical for both types of words in each group, as suggested by [56]. The changes in *S* as a function of the average number of edges connected with each node, which is called the degree, or *K*, were determined, and the optimal value of *K* was determined so that the difference between the two types of words was maximized.

### Statistical analysis

The response times (RTs) and error rates were subjected to mixed-design, repeated-measures analysis of variance (ANOVA) with word type (old/new) as a within-subject factor and group (the schizotypal trait group and the normal control group) as a between-subjects factor. Greenhouse-Geisser corrections were employed when the sphericity assumption was not satisfied, and the corrected *p*-values and degrees of freedom were reported. The variables that showed significant main effects were further analyzed with *post hoc* pairwise comparisons using a paired-sample *t*-test, with *p*-values corrected by the false discovery rate (FDR) method to address the multiple comparisons problem.

Based on the visual inspection of the grand average and individual ERSP maps, five 100-ms time intervals for the period from 250 to 750 ms after stimulus onset and four regions of interest (ROIs), i.e., frontal (Fz, F3, F4), central (Cz, C3, C4), parietal (Pz, P3, P4), and occipital (Oz, O1, O2), were selected. The mean amplitude of the spectral power in the theta band (4–8 Hz) in each interval and ROI was calculated and analyzed using mixed-design, repeated-measures ANOVA with ROI (frontal, central, parietal, and occipital areas) and word type as the within-subject factors and group as a between-subjects factor.

Five 100 ms intervals were selected for statistical analysis of the TPLVs from 250 to 750 ms after stimulus onset, as performed for the ERSPs. The mean number of significant connections at each interval was calculated and analyzed using mixed-design, repeated-measures ANOVA with word type as a within-subject factor and group as a between-subjects factor.

Based on the dual-process model of recognition memory, we focused on the network characteristics during two temporal intervals devoted to ‘familiarity’ and ‘recollection’ processing (i.e., 250–550 and 550–750 ms, respectively). The small-worldness, *S*, which was calculated from a TPLV graph at the optimal degree, *K*, was analyzed using mixed-design, repeated-measures ANOVA with word type as a within-subject factor and subject group as a between-subjects factor.

Possible correlations between the EEG measures, including TBA, TPLV, and *S*, and the behavioral measures were explored within each group by Pearson’s correlation coefficient. In particular, we investigated the correlation between the old/new difference in the EEG measures and the old/new difference in the behavioral measures to elucidate the association of the EEG measures with recognition memory function, i.e., the old/new effect.

## Results

### Behavioral results

A significant interaction effect for group × word type was observed for response time (*F*(1,32) = 7.18, *p*<0.05). The control group responded more rapidly to the old words than to the new words (538.31 ms vs. 551.42 ms), whereas the schizotypal trait group responded significantly more slowly to the old words than to the new words (565.17 ms vs. 533.61 ms). A main effect of word type and an interaction between group × word type were found for error rate (*F*(1,32) = 4.08, *p*<0.05). The old words elicited more errors than the new words. The control group exhibited no difference in the error rate between the old and new words, whereas the old words elicited more errors than the new words in the schizotypal trait group.

### Event-related spectral perturbation (ERSP) in the theta band

**Fig 1:**
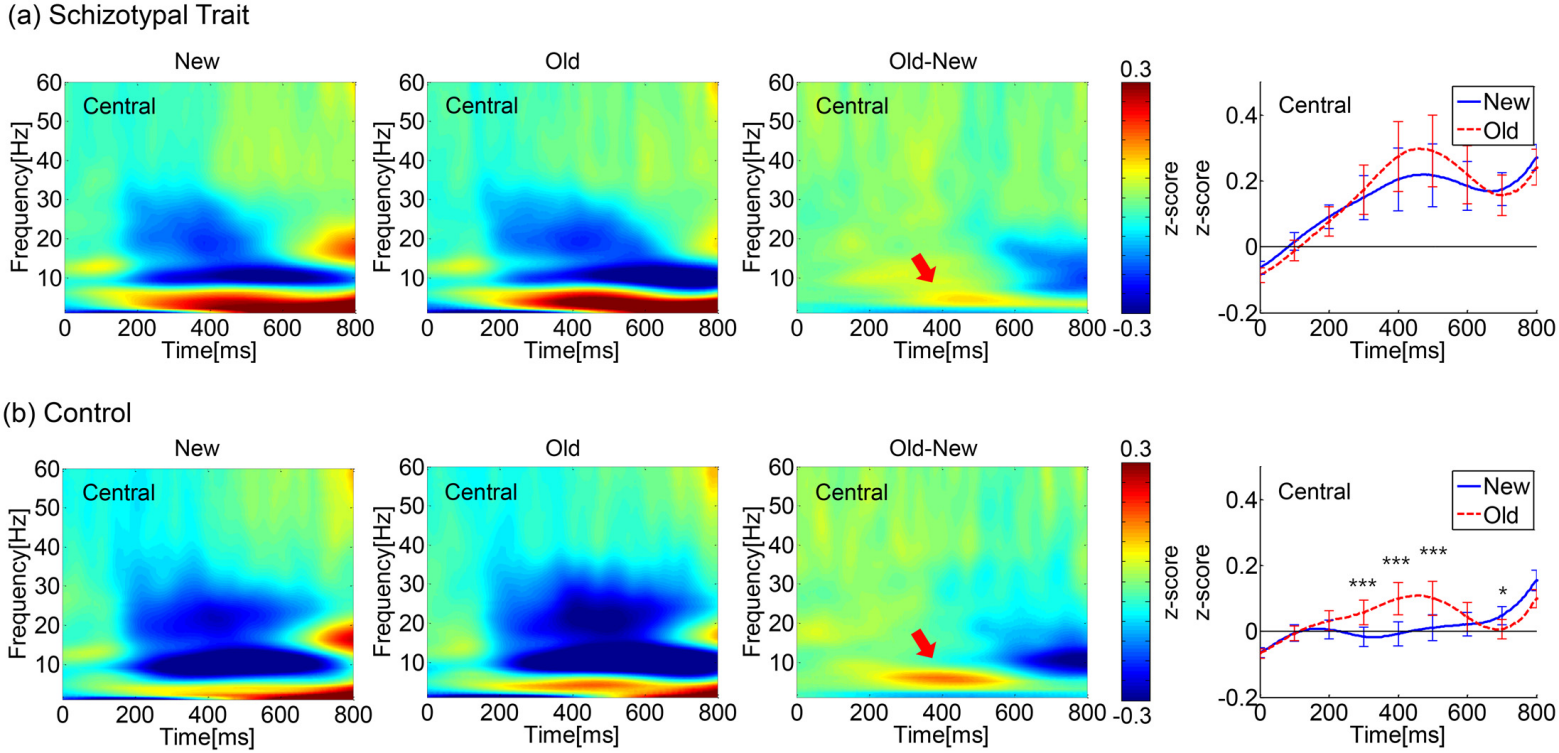
Event-related spectral perturbation (ERSP) in the theta band. Time-frequency activation patterns for the 1–60 Hz signal for the new and old words and the contrast between them (old-new) for the (a) schizotypal trait group and (b) control group. Each ERSP map was obtained by averaging the ERSPs at three central electrodes (C3, Cz, and C4). The rightmost panels show the time courses of the averaged induced TBA (averaged from 4–7 Hz) for the new and old words (***: *p*<0.005, *: *p*<0.05, *post hoc* pairwise comparison with FDR correction). The induced TBA started to increase at ~100 ms and peaked at ~400–500 ms post-stimulus. Prominent differences in the induced TBA between the old and new words were observed throughout the five 100 ms time intervals over the period from 250 to 750 ms in both groups. However, the difference in TBA between the old and new words was altered at ~650 ms. (The old words elicited higher TBA at 250–650 ms but lower TBA at 650–750 ms compared to the new words.) As the contrast ERSP maps (3^rd^ columns) show, during the 250–550 ms interval, a prominent old/new difference was observed in both groups. However, the difference seemed to be much weaker for the schizotypal trait group, as indicated by the red arrows.

**Fig 2:**
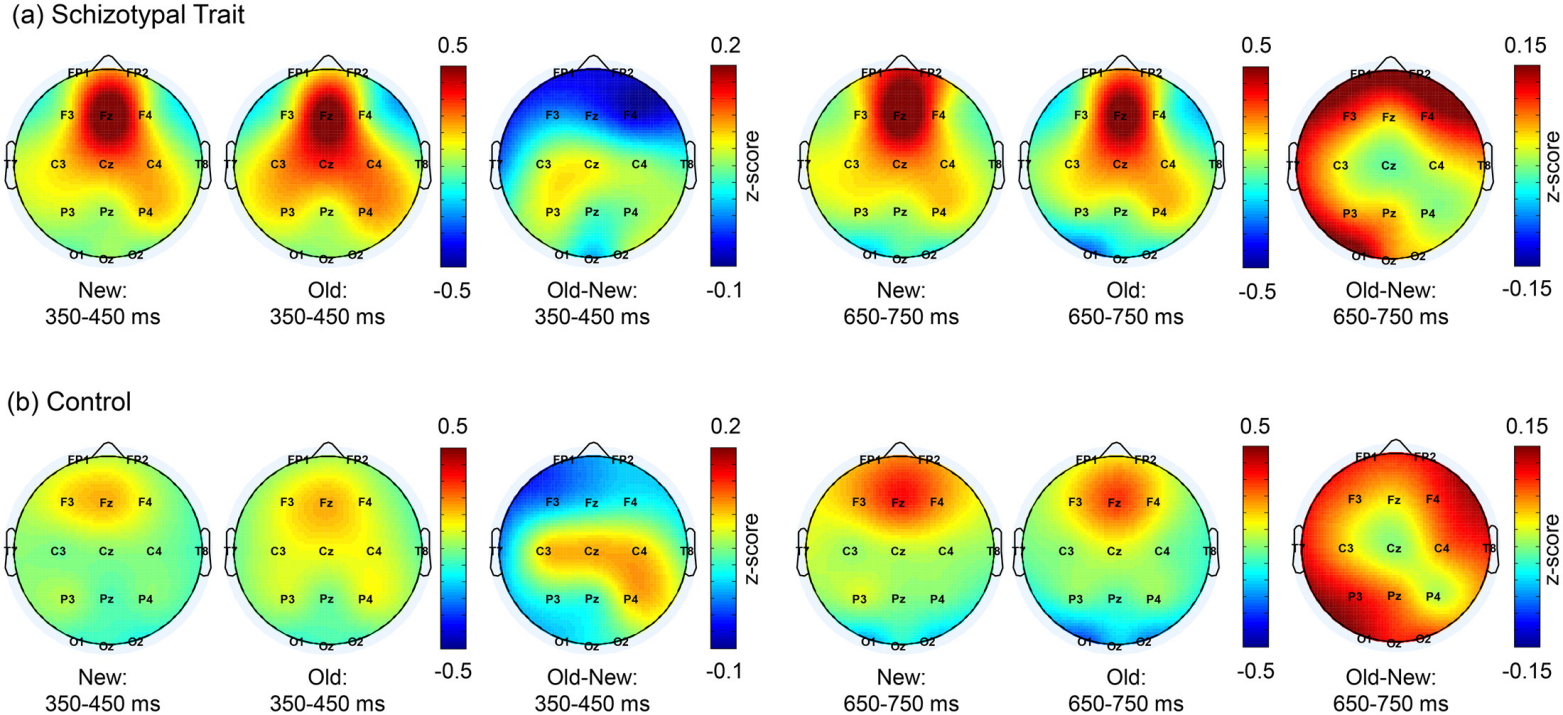
Topographical distributions of theta-band activity (TBA). Topographical maps of induced TBA for the new and old words and the contrast between them (old-new) during the 350–450 and 650–750 ms intervals in the (a) schizotypal trait and (b) control groups. The induced TBA was centered in the frontocentral region. The largest old/new difference was observed in the frontocentral area during an early period (350–450 ms) and in the frontal and parietal areas during later periods (650–750 ms).

The ERSP maps and time courses of the induced TBA averages for three central electrodes (C3, Cz, and C4) are shown in Fig 1. The spatial distribution of the induced TBA during the 350–450 ms and 650–750 ms intervals is shown in Fig 2.

Significant main effects were found for word type and ROI. The old words elicited significantly higher theta power than did the new words at 350–450 ms (*F*(1,32) = 7.31, *p* = 0.011) but lower theta power than did the new words at 650–750 ms (*F*(1,32) = 16.37, *p*<0.001). With respect to the ROIs, the TBA was highest in the frontal area during all time intervals. The smallest TBA was observed in the parietal area during the 250–350 ms interval (*F*(3,96) = 4.79, *p* = 0.009, *ε* = 0.736) and in the occipital area during the 350–450 ms (*F*(3,96) = 13.34, *p*<0.001, *ε* = 0.725), 450–550 ms (*F*(3,96) = 29.05, *p*<0.001, *ε* = 0.733), 550–650 ms (*F*(3,96) = 45.38, *p*<0.001, *ε* = 0.737), and 650–750 ms intervals (*F*(3,96) = 63.97, *p*<0.001, *ε* = 0.731).

An interaction effect of word type × ROI was observed during all time intervals (*F*(3,96) = 5.39, *p* = 0.005, *ε* = 0.752, *F*(3,96) = 6.97, *p* = 0.002, *ε* = 0.655, *F*(3,96) = 7.01, *p* = 0.003, *ε* = 0.579, *F*(3,96) = 6.20, *p* = 0.005, *ε* = 0.593, and *F*(3,96) = 4.78, *p* = 0.009, *ε* = 0.740 for 250–350, 350–450, 450–550, 550–650 and 650–750 ms, respectively).

Although neither a significant interaction between word and group nor a main effect of group was observed, a stronger old/new difference was observed in the control group; as the time-frequency map shows, the old/new difference in the TBA was much larger for the controls (red arrows, third column of Fig 1). We performed a *post hoc* pairwise comparison between the old and new word types in the central regions within each group at each temporal point and found significant differences between the two word types only in the controls during the 250–550 ms and 650–750 ms periods (denoted in the rightmost column of Fig 1). As shown in the rightmost panels of Fig 1, in the control group, the old words elicited higher TBA than the new words in the central area during the 250–550 ms period (*t*(16) = -4.41, *p* = 0.002, *t*(16) = -5.00, *p* = 0.001, and *t*(16) = -4.01, *p* = 0.004, FDR corrected, for 250–350, 350–450, and 450–550 ms, respectively). However, in the schizotypal trait group, a significant old/new difference was not found at any interval. During the 550–750 ms interval in the control group, the old words elicited lower TBA than the new words in the occipital area at 550–650 ms (*t*(16) = 3.25, *p* = 0.02) and in all areas at 650–750 ms (*t*(16) = 2.14, *p* = 0.048, *t*(16) = 2.51, *p* = 0.031, *t*(16) = 2.79, *p* = 0.026, and *t*(16) = 5.24, *p*<0.001, FDR corrected, for frontal, central, parietal, and occipital areas, respectively). However, in the schizotypal trait group, a similar effect was found only in the occipital area during the 650–750 ms interval (*t*(16) = 2.81, *p* = 0.050, FDR corrected).

The correlation between TBA and behavioral responses was investigated within each ROI in each group for the 350–450 ms period, when the old/new difference in TBA was largest. Significant correlations between the old/new difference in TBA in frontal regions and the old/new difference in error rates were observed in the control group (*r* = -0.50, *p* = 0.040) but not in the schizotypal trait group (*r* = -0.36, *p* = 0.16).

Table 1 shows the mean amplitude of induced TBA for the old and new words in both groups at each time interval.

**Table 1:** The mean amplitude of the theta-band activity (TBA) induced by new and old words in the schizotypal trait and control groups.

**Schizotypal trait (*n* = 17)**										
	**250–350 ms**		**350–450 ms**		**450–550 ms**		**550–650 ms**		**650–750 ms**	
**ROI**	New	Old	New	Old	New	Old	New	Old	New	Old
**F**	0.25 (0.25)	0.18 (0.19)	0.29 (0.32)	0.24 (0.28)	0.32 (0.33)	0.26 (0.30)	0.32 (0.30)	0.24 (0.28)	0.33 (0.26)	0.23 (0.24)
**C**	0.15 (0.28)	0.18 (0.32)	0.20 (0.40)	0.27 (0.45)	0.21 (0.40)	0.29 (0.46)	0.18 (0.31)	0.22 (0.37)	0.18 (0.21)	0.16 (0.26)
**P**	0.09 (0.22)	0.11 (0.24)	0.10 (0.30)	0.15 (0.34)	0.09 (0.31)	0.15 (0.37)	0.08 (0.27)	0.09 (0.32)	0.11 (0.20)	0.07 (0.23)
**O**	0.18 (0.19)	0.18 (0.21)	-0.02 (0.11)	0.00 (0.15)	-0.13 (0.12)	-0.12 (0.15)	-0.15 (0.15)	-0.20 (0.15)	-0.11 (0.18)	-0.20 (0.15)
**Control (*n* = 17)**										
	**250–350 ms**		**350–450 ms**		**450–550 ms**		**550–650 ms**		**650–750 ms**	
**ROI**	New	Old	New	Old	New	Old	New	Old	New	Old
**F**	0.11 (0.20)	0.10 (0.19)	0.14 (0.28)	0.14 (0.25)	0.20 (0.32)	0.18 (0.28)	0.24 (0.29)	0.20 (0.24)	0.31 (0.22)	0.24 (0.18)
**C**	-0.01 (0.13)	0.06 (0.16)	-0.01 (0.15)	0.10 (0.21)	0.01 (0.17)	0.10 (0.22)	0.02 (0.15)	0.04 (0.18)	0.05 (0.12)	0.01 (0.12)
**P**	-0.01 (0.13)	0.04 (0.12)	0.00 (0.15)	0.05 (0.16)	0.00 (0.16)	0.03 (0.18)	0.00 (0.14)	-0.01 (0.17)	0.04 (0.12)	-0.02 (0.12)
**O**	0.12 (0.13)	0.14 (0.16)	-0.10 (0.15)	-0.08 (0.15)	-0.20 (0.16)	-0.21 (0.17)	-0.21 (0.16)	-0.27 (0.16)	-0.14 (0.17)	-0.26 (0.16)

All values are represented as the mean, with the standard deviation in parentheses. The mean values were obtained by averaging the theta power amplitude at each ROI (F: frontal, C: central, P: parietal, O: occipital areas) during each time period.

### Theta-band PLV (TPLV) and graph theory analysis

**Fig 3:**
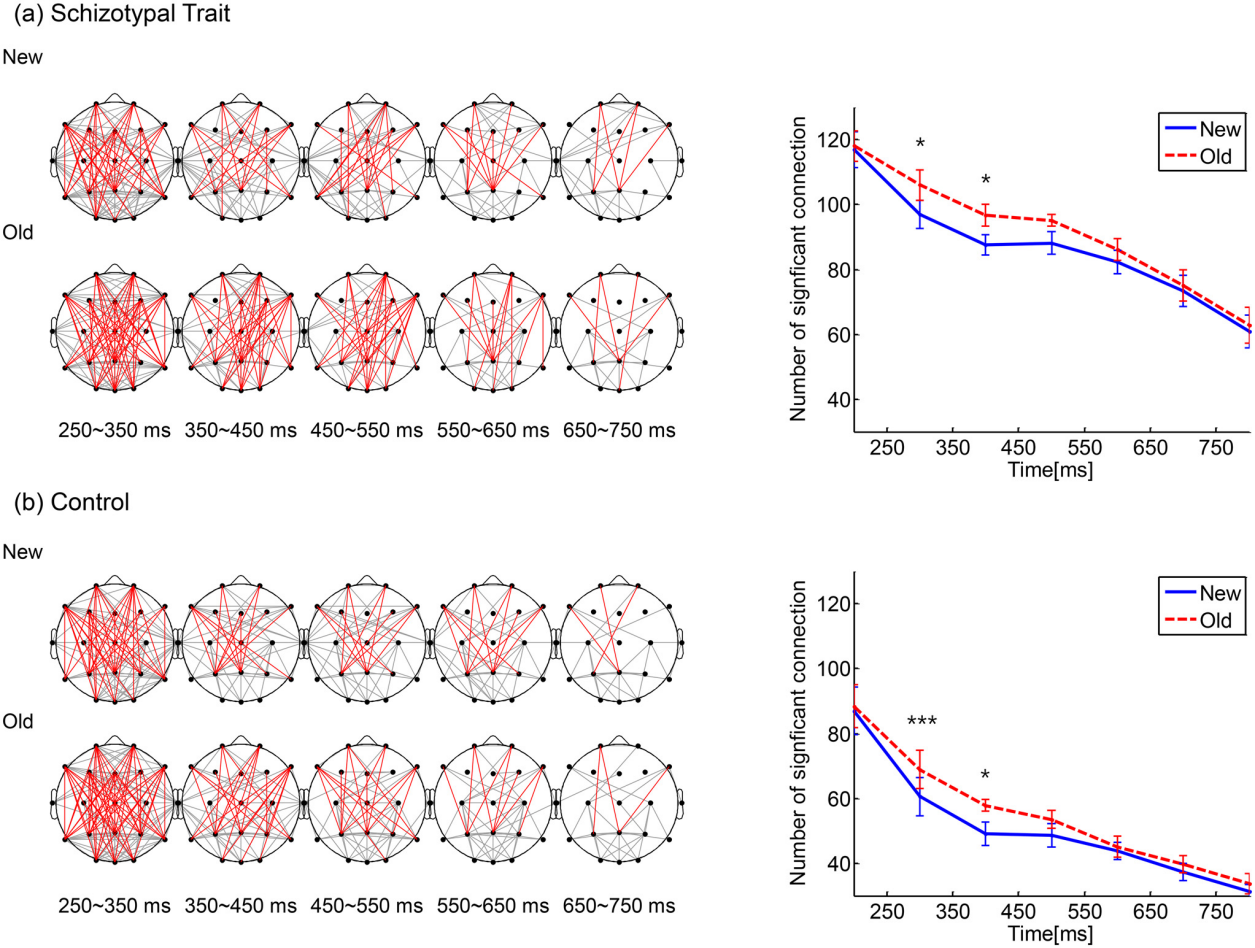
Inter-regional theta-band PLVs (TPLVs). Spatiotemporal pattern of TPLVs for the new and old words for (a) the schizotypal trait group and (b) the control group. Red: significant TPLVs between anterior and posterior regions (the Fp1, Fp2, F7, F3, Fz, F4, F8, T5, P3, Pz, P4, T6, O1, Oz, and O2 electrodes were used); gray: among other regions. The time (in ms) written below each map indicates the 100 ms interval from which the TPLV was calculated. The right panels show the time courses of the mean number of significant connections across subjects within each group (error bar: standard error). In both groups, the most apparent old/new difference in the TPLVs occurred during the 250–550 ms interval, when the old words elicited higher TPLVs than the new words (*: *p*<0.05, ***: *p*<0.005).

Fig 3 shows the temporal evolution of the inter-regional TPLVs (averaged across subjects within each group). Significant TPLVs occurred primarily between the anterior and posterior regions for both word types in both groups. Significant anterior-posterior TPLVs are denoted with red lines. (The Fp1, Fp2, F7, F3, Fz, F4, F8, T5, P3, Pz, P4, T6, O1, Oz, and O2 electrodes were used.) Significant TPLVs among other regions are designated with gray lines. In both groups, the most apparent old/new difference in TPLVs was observed during the 250–550 ms interval (Fig 3, right panels), when the old words elicited higher TPLVs than the new words.

Significant main effects were found for word type and group. The old words elicited significantly more TPLV connections than the new words at 250–550 ms (*F*(1,32) = 13.83, *p* = 0.001, *F*(1,32) = 14.91, *p* = 0.001, and *F*(1,32) = 5.45, *p* = 0.026 for 250–350, 350–450, and 450–550 ms, respectively). With respect to group, the number of TPLV connections was significantly greater in the schizotypal trait group during all time intervals (*F*(1,32) = 27.09, *p*<0.001, *F*(1,32) = 113.68, *p*<0.001, *F*(1,32) = 141.66, *p*<0.001, *F*(1,32) = 96.44, *p*<0.001, and *F*(1,32) = 49.06, *p*<0.001 for 250–350, 350–450, 450–550, 550–650 and 650–750 ms, respectively). The interaction effect of word type × group was not significant during any of the time intervals. In addition, the TPLV connections and behavioral measures within each group were not significantly correlated during any of the time intervals.

**Fig 4:**
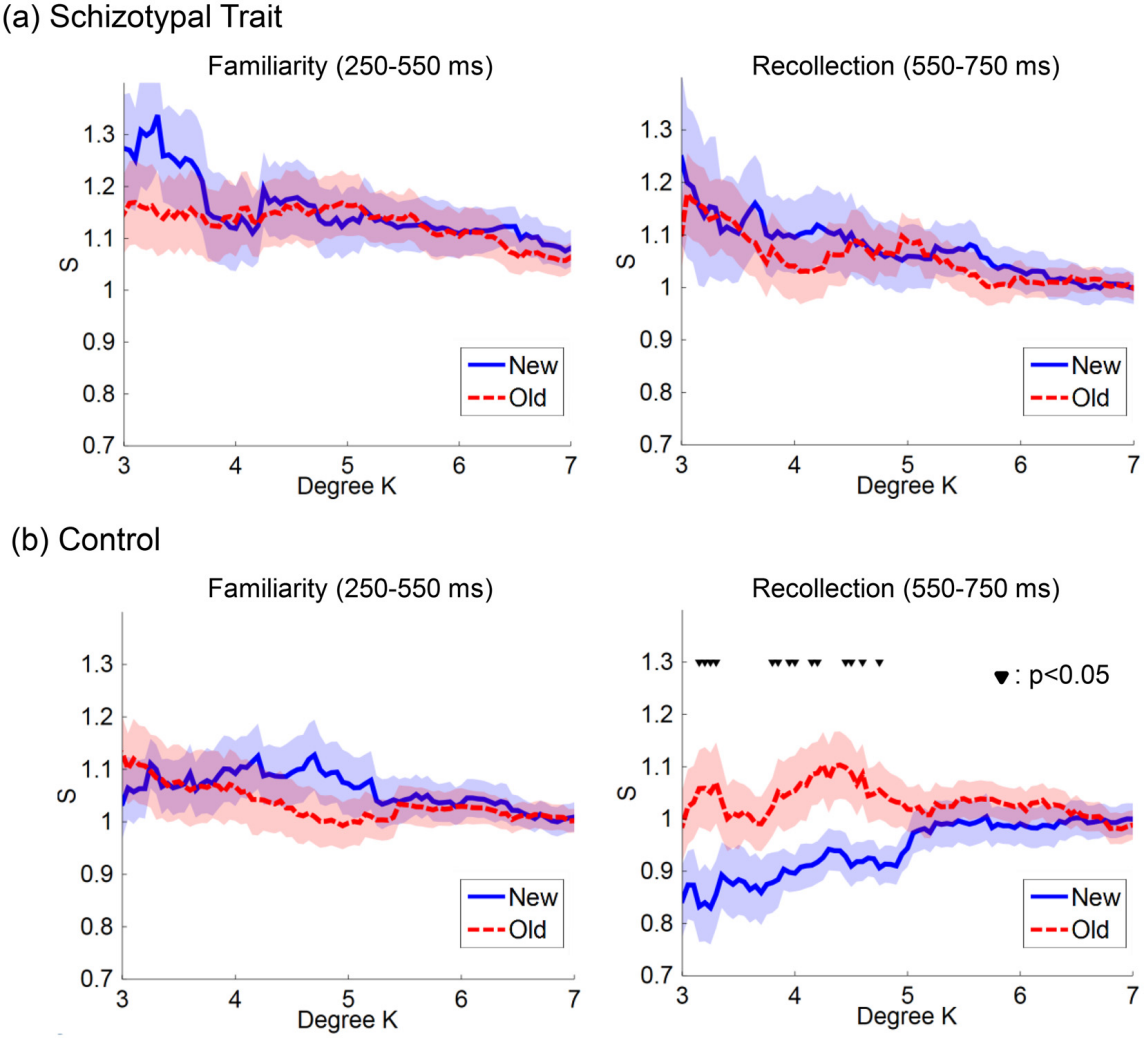
The network small-worldness (*S*) of the theta-band phase locking value (TPLV) maps as a function of degree (*K*). (a) The schizotypal trait and (b) control groups. Blue solid line: new word. Red dotted line: old word. Shaded area: standard error. Based on the dual-process model of recognition memory, we focused on networks during the two temporal intervals related to ‘familiarity’ and ‘recollection’ processing (i.e., the 250–550 and 550–750 ms intervals, respectively). Within each group, the *S* values for the old and new words were statistically compared at each *K* from 3 to 7. During the 250–550 ms period, both groups did not reveal that any *S* was significantly higher for the old words than for the new words at several degrees (*K*s; between 3 and 5) only in the control group (▼: *p*<0.05 by paired-sample *t*-test). The optimal *K* was defined as 3.3, which showed the largest difference between the two types of words. However, the schizotypal trait group did not show a significant old/new effect in *S* at any *K* during the 550–750 ms period.

Fig 4 shows the network small-worldness, *S*, as a function of degree, *K*, at two time intervals within each group. During the 250–550 ms period, neither group exhibited a significant old/new difference in *S* at any *K*. During the 550–750 ms period, the *S* was significantly higher for the old words than for the new words at several degrees (*K*s) in the control group. The optimal *K* was defined as 3.3, which showed the largest difference between the two word types (*t*(16) = -2.66, *p* = 0.017 by paired-sample *t*-test). However, the schizotypal trait group did not show a significant old/new effect in *S* at any *K* during the 550–750 ms period.

A significant main effect of word type or group was not observed at this time interval, and neither was an interaction effect for word type × group. However, we observed that the level of old/new difference in *S* explicitly differed between the two groups, as shown in the right panels of Fig 4; thus, we performed a *post hoc* pairwise comparison between the two word types within each group. The *S* was significantly higher for the old words than for the new words only in the control group (*t*(16) = -2.66, *p* = 0.017 by paired-sample *t*-test). The correlations between the network small-worldness and the behavioral measures within each group were not significant during the 550–750 ms interval.

## Discussion

Our results suggest that explicit memory deficits in subjects with schizotypal traits are associated with abnormal theta-band neuronal synchrony, supporting and extending our recent findings from conventional ERP analysis [20]. We observed the trends of differentiation in theta-band rhythm and inter-regional phase synchrony between the control and schizotypal trait groups. An old/new effect was found in the induced theta-band ERSP in both groups. However, the difference between the old and new tended to be elevated in normal controls. The tendency of elevated old/new effect in normal controls was observed in anterior-posterior theta-band phase synchrony as well.

As described in the result, the interaction between word type and group were not significant, and the tendency of weaker old/new effect in schizotypal trait group was found by pairwise comparisons between word types within each group. Hence, the abnormality in theta-band ERSP and inter-regional phase synchrony associated with schizotypal trait should be characterized further in the future studies. Nevertheless, it would be meaningful in that we provided the possibility for the first time. We expect that these results may provide valuable insight into the mechanisms of memory dysfunction due to schizophrenia.

We observed the old/new effect in the theta-band rhythm and inter-regional phase synchrony in the theta-band during the 250–750 ms interval, and the effects were smaller in the schizotypal trait group than in the normal control group. That is, based on the pairwise comparisons between the old and new word types for each group, the old/new effect in induced TBA was found throughout the 250–750 ms interval in the control group, but it was apparent for only limited temporal intervals in the schizotypal trait group. It is well recognized that theta-band rhythm and synchrony play important roles in memory [57–59]. For example, theta rhythm is selectively increased during the encoding/retrieval of new information [57], and the coupling of theta-band activity within the MTL and rhinal-hippocampal interactions contributes to successful memory formation [58, 59]. The role of the theta rhythm in frontal areas in the memory process has also been demonstrated [32, 60]. In addition to being observable in the ERP, the old/new effect was also observable in the theta-band activity [61]. The relationship between explicit verbal memory performance and theta power has also been reported [38]. Baving et al. showed that the old/new effect in ERPs was lower in schizophrenic patients than in normal controls [26]. In addition, theta rhythm abnormalities in schizophrenic patients have been observed during resting-state and memory functions [39, 62, 63]. Our results regarding the old/new effect on theta power and its decrease in the schizotypal trait group are in accordance with these previous findings. Furthermore, we observed that comparable old/new effects and inter-group differences in the old/new effect also occurred in inter-regional phase synchrony. The old/new effect in inter-regional TPLVs was noteworthy only for normal controls during limited time intervals (the 550–750 ms intervals, as shown by graph theory analysis). The implications of phase synchrony in memory processes have been acknowledged and are attributed to its facilitation of neural communication and promotion of neural plasticity [57, 59, 64].

The most crucial difference between the findings from our previous ERP study and those of the present study is that the temporal periods showing old/new effect abnormalities were altered for the theta rhythm. The meaning of the temporal period of the abnormalities may be understood based on the dual process model of recognition memory, which suggests that performance on recognition memory tests reflects the assessment of stimulus “familiarity” and the “recollection” of details pertaining to a previous episode [29, 30]. These models assume that familiarity processing, which is automatic, occurs earlier and that more controlled recollection processing occurs later, during recognition memory. ERP studies have provided evidence in support of this model, such as a mid-frontal old/new effect in early periods (300–500 ms) and a left parietal old/new effect in a later period (500–700 ms), which represent familiarity and recollection, respectively [65–67]. Neuroimaging studies have demonstrated that the brain regions involved in the familiarity and recollection aspects of recognition memory are different to some extent. Structures in the MTL, including the hippocampus [68] and the parietal lobe [69], are considered to play important roles in recollection, whereas the prefrontal cortex is crucial in familiarity processing [70, 71].

In previous studies, the old/new effect was reduced during the 500–700 ms interval but not in the 300–500 ms interval (based on ERP studies of recognition memory) in schizophrenic patients or non-clinical individuals with schizotypal traits [20, 25]. This finding led to the conclusion that recognition memory deficits in schizophrenia spectrum disorders, including schizophrenia, originate from deficits in recollection/retrieval. However, contrary to this conclusion, our current theta-band rhythm results also showed reduced old/new effects during earlier intervals (250–550 ms). This finding may indicate that the deficits in explicit verbal memory associated with schizotypal traits are caused by abnormal familiarity processing and by abnormal recollection processing. These results also suggest the limitation of conventional averaged ERP analysis and the necessity/usefulness of rhythm analyses.

In the current study, the theta rhythm was highest in the mid-frontal region throughout performance of the task for both groups and word types, and the inter-group difference in the old/new effect in theta activity was most evident in the bilateral frontal areas (Fig 3). These results are in line with the acknowledged importance of frontal theta rhythm in explicit memory processing and the well-known deficit in synaptic-level frontal lobe function and its influence on the theta rhythms associated with schizophrenia spectrum disorders [39, 61, 72, 73]. The theta rhythm reflected in the EEG results from hippocampal inhibitory theta rhythm, which is transferred to the cortex by limbic-hippocampal-cortical re-entrant loops. It was suggested that the reduction of prefrontal dopamine receptor activation in schizophrenia, together with decreased NMDA and GABA signaling, may lead to unfocused cortical activation and reduced recurrent inhibition, which in turn results in the reduction of the signal-to-noise ratio of the theta-band activity in schizophrenia [73]. In line with these hypotheses, the old/new effect in the TBA was diminished, and it was not correlated with the behavioral measures in the schizotypal trait group.

Although the detailed mechanisms underlying frontal theta rhythm generation are not fully understood, frontal theta rhythms are associated with the encoding of information [74] and reinforcement learning [75]. Frontal theta power increases during information encoding to predict the subsequent successful recall of information [76]. A correlation between frontal theta oscillations and working memory load has also been reported [31, 60]. Thus, theta activity seems to be crucial in the explicit memory tasks in our study, and the reduced old/new effect in the TBA in the schizotypal trait group during the 250–550 ms period may indicate that these individuals may have difficulties in familiarity processing during recall.

The old/new difference in inter-regional phase synchrony was most apparent during the 250–550 ms interval in both groups, and higher connectivity for the old words was observed. Skinner and Fernandes (2007) suggested the importance of the network consisting of frontal and parietal areas in recognition memory; these areas are involved in the retrieval/encoding and storage of information, respectively [77]. In line with this suggestion, we observed significant TPLVs between the anterior and posterior regions, including frontal and parietal areas. However, due to the inherent limitation of the spatial resolution, the exact location of the greatest connectedness could not be identified by the surface EEG-based method used in our study. Phase synchrony analysis at the level of cortical current sources may be useful to alleviate this problem, and thus, this seems worth trying in further studies [78, 79]. Nevertheless, the sensor-level analysis adopted in our study provides useful information for comparisons between conditions and/or subject populations [80, 81]. For example, a significant increase in TPLV during 250–550 ms was observed in schizotypal trait group as compared to normal controls regardless of word type. Memory processing has been thought to be sensitive to precise modulations of theta-band synchronization in widely distributed brain regions [64, 82]. Accordingly, the increased TPLVs observed in our study may reflect interference in the modulation of theta activity during memory retrieval, which in turn results in recognition memory dysfunction. This hypothesis is supported by a previous study that showed that resting-state functional connectivity in the theta band was elevated and correlated with verbal memory deficits in schizophrenia patients [83].

Furthermore, surface EEG-based inter-regional phase synchrony analysis could provide additional valuable information when combined with graph theory measures of network patterns [56, 84]. For example, by pairwise comparisons between old and new word types for each group, the differences in theta-band inter-regional phase synchrony between the old and new words was found only in the control group from the network small-worldness during the 550–750 ms interval, whereas the inter-group comparison was difficult to interpret using visual inspection of the phase synchrony map alone. This finding may indicate that individuals with schizotypal traits have difficulty in both the familiarity and recollection processing components of recognition memory, resulting from dysfunction in coordinated network activity. The graph theory analysis revealed that the TPLV networks corresponded to a small-world network and that the measure of ‘small-worldness’ was greater for the old words than for the new words only in the normal controls. It can be hypothesized that the functional cortical network for word recognition is more efficient in terms of inter-regional communication in the normal controls. It would be meaningful to perform a similar analysis in schizophrenia patients because several previous studies have shown that small-worldness was disrupted in schizophrenia patients during resting-state and cognitive tasks [85, 86].

In summary, we found that the deficits in explicit verbal memory in individuals with schizotypal traits were associated with abnormalities in theta-band rhythm and inter-regional synchrony. The old/new difference was diminished in the schizotypal trait group for both theta rhythm and inter-regional phase synchrony. This finding supplements our previous results of reduced old/new effects in the ERP waveform during a later period devoted to recollection because the trends suggesting inter-group differences in theta power and TPLVs were found during an earlier period, which implies that familiarity processing and recollection processing were impaired in the schizotypal trait group. Our results suggest that explicit memory deficits shown in schizophrenia patients can also be observed even in non-clinical individuals with psychometrically defined schizotypal traits. Therefore, these results support previous findings suggesting that explicit memory dysfunction is a core problem associated with schizophrenia.

## Supporting Information

S1 File Numerical values of the induced theta-band activity (TBA) shown in Table 1. The TBA for each individual was obtained by averaging the amplitude of the theta-band power at each ROI (F: frontal, C: central, P: parietal, O: occipital areas). Five Excel worksheets are included, corresponding to the five temporal periods: 250–350 ms, 350–450 ms, 450–550 ms, 550–650 ms, and 650–750 ms.
(XLSX)

S2 File Numerical values of the graph theory measures (at degree, *K* = 3.3) of the theta-band phase locking value (TPLV) pattern during the 550–750 ms period described in Fig 4. One Excel worksheet is included, corresponding to the temporal period of 550–750 ms.
(XLSX)
